# Pleiotropic effects of oxytocin receptor polymorphisms: influencing striatocortical connectivity in bipolar disorder

**DOI:** 10.1186/s40345-025-00393-8

**Published:** 2025-08-31

**Authors:** Shyh-Yuh Wei, Huai-Hsuan Tseng, Hui Hua Chang, Po See Chen

**Affiliations:** 1 Department of Psychiatry, National Cheng Kung University Hospital, College of Medicine, National Cheng Kung University, Tainan, Taiwan; 2 Institute of Behavioral Medicine, College of Medicine, National Cheng Kung University, Tainan, Taiwan; 3 Institute of Clinical Pharmacy and Pharmaceutical Sciences, College of Medicine, National Cheng Kung University, Tainan, Taiwan; 4 School of Pharmacy, College of Medicine, National Cheng Kung University, Tainan, Taiwan; 5 Department of Pharmacy, National Cheng Kung University Hospital, College of Medicine, National Cheng Kung University, Tainan, Taiwan; 6 Department of Pharmacy, National Cheng Kung University Hospital, Dou- Liou Branch, Yunlin, Taiwan

**Keywords:** Childhood trauma, Corticostriatal, Functional connectivity, rs53576, rs2228485

## Abstract

**Background:**

Oxytocin (OXT), a neuropeptide involved in social behaviors and emotions, exhibits bidirectional effects depending upon positive or negative environments. Our previous report highlighted dysregulation of OXT on striatocortical functional connectivity (FC) in bipolar disorder (BD) patients. We hypothesized that: (1) in healthy controls (HC), carriers of a “sensitive” *OXTR* allele would show altered FC, particularly in association with childhood trauma; and (2) this gene–brain relationship would be fundamentally altered or reversed in BD patients, reflecting a gene–disease interaction.

**Method:**

Thirty-nine BD patients and 32 age-matched HC underwent resting-state functional MRI and blood sampling for genotyping and plasma OXT level assessment.

**Results:**

BD patients, compared to HC, demonstrated elevated plasma OXT levels and higher scores in childhood trauma. Gene–disease interactions were observed in the striatocortical circuitry with *OXTR* rs53576 and rs2228485, with greater robustness in rs2228485. In HC, the rs2228485 AA homozygotes showed enhanced striatocortical FC with the sensory association and limbic areas, which were correlated with the childhood trauma. Conversely, alterations in ventral striatocortical FC were reversed among BD patients, with hypo-FC in AA homozygotes and hyper-FC in G-allele carriers.

**Conclusions:**

These findings highlight a gene–disease interplay, suggesting that individuals carrying the “sensitive” allele may exhibit context-dependent alterations in salience-related brain networks. Our results identify a potential neural mechanism through which the *OXTR* polymorphism modulates environmental sensitivity, with distinct effects in HC and BD. Childhood trauma may shape striatocortical FC in an *OXTR* genotype-dependent manner.

**Supplementary Information:**

The online version contains supplementary material available at 10.1186/s40345-025-00393-8.

## Introduction

Oxytocin (OXT) is a neuropeptide critical to social behaviors and emotions (Quintana et al. 2017). Furthermore, oxytocin receptor (*OXTR*) single-nucleotide polymorphism (SNP) may influence human socio-emotional behaviors that also related to peripheral OXT (Grinevich, Neumann 2021). The *OXTR* SNP, serving as an innate mechanism conveying differential susceptibility, has been implicated in gene–environment interactions that affect loneliness (van Roekel et al. 2013), depression (McQuaid et al. 2013) and personality (Brüne 2016). Based on previous research, OXT may enhance social salience with bi-directional effects depending on positive or negative environments—that is, promoting prosocial behavior in supportive environments while amplifying sensitivity to threat or rejection in adverse contexts (Tabak 2013).

Previous neuroimaging studies have revealed OXT effects on salience-related resting-state functional connectivity (FC), while *OXTR* SNP may impact the reward system (Seeley et al. 2018). Despite some reports shedding light on the impact of different *OXTR* SNP on social behaviors and psychiatric disorders, the underlying neural mechanisms remain a dark forest (Aspé-Sánchez et al. 2015). Questions persist regarding how resting-state FC changes relate to behavior, necessitating further investigations (Seeley et al. 2018). Imaging genetics studies, integrating multimodal biological measures that encompass genetic, neurochemical, neuroimaging, and behavioral factors, aid in identifying intermediate phenotypes, potentially leading to a better understanding in the gene–disease interactions (Ching et al. 2020; Pereira et al. 2017).

A growing body of literature explores the impact of *OXTR* SNP on autism spectrum disorder (Aspé-Sánchez et al. 2015; Hernandez et al. 2017; LoParo, Waldman 2015), while some *OXTR* SNPs may be associated with negative affects beyond autism (Kazantseva et al. 2020; Sicorello et al. 2020; Wu et al. 2020). Among *OXTR* SNP, rs53576, associated with socio-emotional behaviors (Meyer-Lindenberg et al. 2011), has central effects influenced by childhood trauma (Malhi et al. 2020). Additionally, we reported that *OXTR* rs53576 modulates interactions between the oxytocinergic and reward systems in healthy controls (HC) (Chang et al. 2014). Notably, rs2228485, a SNP producing a guanine (G)-to-adenine (A) substitution at exon 3 in the human *OXTR* gene, is associated with lower loneliness scores (Lucht et al. 2009) and better ability to read emotional responses in male faces (Lucht et al. 2013). Whether such associations in HC reverse in negative environments or patient populations remains unclear.

Bipolar disorder (BD), with childhood trauma as a major risk factor (Aas et al. 2016), is characterized by emotion dysregulation and social cognitive impairments, including abnormalities in emotional processing (Furlong et al. 2021). BD patients may express increased *OXTR* mRNA in peripheral leukocytes (Iacob et al. 2013) and in postmortem brain tissues (Lee et al. 2018), indicating dysregulation of oxytocinergic signaling. Elevated plasma OXT levels (Lien et al. 2017) and dysregulation of OXT in the striatocortical circuitry have been observed in BD patients (Wei et al. 2020). Given the central role of OXT in social cognition and emotional processing, there is growing interest in using OXT to treat social dysfunction in BD (Quintana et al. 2017). Furthermore, childhood trauma is associated with striatocortical circuitry dysfunction in BD (Hsieh et al. 2021). The striatocortical circuitry is essential in the reward system, with its dysfunction well characterized in BD (Liu et al. 2012; Marchand and Yurgelun-Todd 2010; Stoddard et al. 2016). As the striatocortical circuitry is involved in computing of sensory, motor and limbic information into behavioral and cognitive output (Burke et al. 2017), its impaired function as an amplifier or filter of such information may influence social cognition and emotional processing.

Collectively, this study employed an imaging genetics approach to investigate how two *OXTR* polymorphisms (rs53576 and rs2228485) influence FC within ventral and dorsal striatocortical circuits in both HC and BD patients. We aimed to examine the association between striatocortical FC and OXT levels, to elucidate potential neurobiological mechanisms by which OXT modulates brain connectivity across different genotypes. Additionally, to explore brain mechanisms linking *OXTR* polymorphisms to early life adversity, we examined correlations between striatocortical FC and childhood trauma scores.

We hypothesized that, in healthy controls, carriers of the putative “sensitive” allele would show increased salience-related striatocortical FC, and that FC strength would positively correlate with childhood trauma scores—consistent with the “environmental sensitivity” hypothesis (Tabak 2013). In BD patients, we expected a significant gene–disease interaction, such that the relationship between *OXTR* genotype, FC, and childhood trauma would be disrupted or reversed, given the higher exposure to adverse environments.

## Methods

### Subjects

All participants were of Han Chinese ethnicity, aged between 18 and 70 years, and were native speakers of either Mandarin or Taiwanese. Patients were recruited from the psychiatric outpatient department of National Cheng Kung University Hospital, while HC were recruited from the community via advertisement. An attending psychiatrist assessed all participants using the Chinese version of the Mini International Neuropsychiatric Interview (MINI) (Sheehan et al. 1998), the 17-item Hamilton Depression Rating Scale (HDRS), and the 11-item Young Mania Rating Scale (YMRS). Patient diagnoses were established according to the Diagnostic and Statistical Manual of Mental Disorders, Fifth Edition (DSM-5). We used the Chinese version of the MINI to confirm diagnoses in the BD patient group and the lack thereof in the HC group. A subset of the participants had been included in previously published studies (Hsieh et al. 2021; Tseng et al. 2021; Wei et al. 2020).

Exclusion criteria for all participants were as follows: (1) major psychiatric disorders other than BD in the patient group; (2) a history of head trauma, organic mental disorders, or other neurological conditions; (3) inflammatory diseases, severe surgical conditions, or critical physical illnesses such as acute coronary syndrome, kidney dialysis, or organ transplantation; and (4) pregnancy, breastfeeding, or a positive pregnancy test.

### Experimental design

All participants underwent assessment using the Childhood Trauma Questionnaire (CTQ) (Bernstein et al. 1997) and the UCLA Loneliness Scale (Wu and Yao 2008) to evaluate negative childhood experiences and negative emotions, respectively. Following enrollment in the study, the administration of medications was recorded and adjusted based on clinical manifestations and patient tolerance. Blood samples were collected during the initial examination; however, genotypes of all participants were not known before the scanning session. For detailed information regarding the CTQ, UCLA Loneliness Scale, plasma OXT measurements, image acquisition, and image preprocessing, please refer to our previously published papers (Hsieh et al. 2021; Tsai et al. 2019; Wei et al. 2020).

### Genotyping

Whole blood samples were collected in 4 mL EDTA tubes and stored at 4 °C. Genomic DNA was extracted from each sample using the QIAamp DNA Blood Kit, following the manufacturer’s instructions (QIAGEN, Hilden, Germany). The extracted DNA was then stored at − 80 °C until further analysis. Genotyping was performed using commercial TaqMan SNP assays (Applied Biosystems, Foster City, CA). Polymerase chain reaction (PCR) amplification and dissociation were conducted, and fluorescence signals were recorded using the ABI 7900HT Fast Real-Time PCR System (Applied Biosystems, Foster City, CA). The system automatically computed the negative derivative of the fluorescence change. SNP genotypes were determined using the StepOne software and verified manually. If discrepancies arose, the analysis was repeated. To ensure objectivity, genotyping was assigned independently by technicians who were blinded to participants’ personal information.

### Definition of ventral and dorsal striatum seed and functional connectivity maps

The ventral ([11, 11, 1] and [-8, 12, 1]) and dorsal ([± 13, 15, 9]) striatum seed regions (3-mm radius) were defined based on previously published literature (Di Martino et al. 2008) and centered at MNI coordinates. For each participant, the mean time-series activity within the seed regions was extracted, and FC maps were generated. The resulting individual-level FC maps were then subjected to Fisher’s r-to-z transformation to obtain z-maps for second-level group analyses.

### Statistical analyses

The statistical analyses were performed using SPSS Statistics 20.0 (SPSS Inc., Chicago, IL), with significance set at *p* < 0.05 (two-tailed). The Hardy-Weinberg equilibrium of the *OXTR* genotype distribution within each group, the interaction between rs53576 and rs2228485 polymorphisms, and genotype distribution differences between groups were evaluated using the chi-square test. A two-way analysis of variance (ANOVA) was conducted to examine the main effects of group (BD vs. HC) and *OXTR* genotype (AA homozygotes vs. G allele carriers), as well as their interaction, on age, plasma OXT level, CTQ score, YMRS score, HDRS score, and loneliness.

### Image analysis

A mixed-effects factorial design model with two factors (genotype and group) was applied to analyze FC maps using SPM12 (Wellcome Trust Centre for Neuroimaging, London, https://www.fil.ion.ucl.ac.uk/spm/). Statistical maps were generated to examine FC differences across the following comparisons: (1) between-group comparisons within each genotype, (2) between-genotype comparisons within each group, and (3) gene–disease interactions. Significance was set at an uncorrected voxel-level threshold of *p* = 0.001, followed by a cluster-level family-wise error rate (FWE)-corrected threshold of *p* = 0.05 for whole-brain multiple comparisons.

For each group, two one-tailed two-sample t-tests were conducted to assess the correlations between FC and either plasma OXT levels or CTQ scores for each genotype. Demeaned values (in SPM) were entered as regressors to identify brain regions showing positive or negative correlations with FC in BD patients and HC. The statistical threshold was set at *p* = 0.001 (uncorrected) at the voxel level, followed by a cluster-level FWE correction at *p* = 0.05.

Since SPM identifies peak coordinates within a confluent cluster, multiple peaks may correspond to different brain regions or Brodmann areas. One representative peak per region or area was reported. For 3D visualization, MRIcroGL (Department of Psychology, University of South Carolina, http://www.mccauslandcenter.sc.edu/mricrogl) was utilized.

### Sensitivity analysis

Because different mood states of the BD patients may result in heterogeneity and influence the results, we conducted a restrictive analysis by including only euthymic BD patients (scores of less than 7 on the HDRS and YMRS).

## Results

### Demographic data and baseline information

Thirty-nine BD patients and 32 age-matched HC of the same Chinese ethnicity were eligible for imaging genetics studies. There was no significant difference in age between the BD patients and HC (Table 1). The medication data for the BD group is as follows: Eleven patients (28.2%) were receiving mood-stabilizer monotherapy (valproic acid or lithium); 22 patients (56.4%) were on a combination of valproic acid and antipsychotics; 5 patients (12.8%) were receiving antipsychotic monotherapy; and one patient (2.6%) was on antidepressant treatment.

**Table 1:** Comparison of demographic data and baseline information between the Oxytocin receptor gene rs2228485 polymorphism and bipolar disorder

	Bipolar patients (*n* = 39)	Controls (*n* = 32)		*p* Value	
			Group	Genotype	Interaction
**Sample size**, **n (%)**					0.145
AA homozygotes	14 (36%)	17 (53%)			
G allele carriers	25 (64%)	15 (47%)			
**Age**,** year**			0.079	0.868	0.171
AA homozygotes	40.14 ± 14.80	31.12 ± 8.82			
G allele carriers	36.68 ± 12.91	35.53 ± 8.77			
**Gender**,** female (%)**			0.259	0.748	
AA homozygotes	11 (79%)	8 (47%)	0.073		
G allele carriers	16 (64%)	10 (67%)	0.864		
**Plasma oxytocin (pg/ml)**			0.040	0.124	0.123
AA homozygotes	229.08 ± 215.58	114.48 ± 72.01			
G allele carriers	131.20 ± 117.42	114.69 ± 82.64			
**Childhood trauma**			< 0.001	0.597	0.984
AA homozygotes	45.50 ± 11.96	34.88 ± 6.97			
G allele carriers	46.92 ± 13.69	36.20 ± 5.02			
**YMRS score**			0.005	0.551	0.477
AA homozygotes	2.07 ± 4.60	0.00 ± 0.00			
G allele carriers	1.32 ± 1.97	0.07 ± 0.26			
**HDRS score** ^a^			< 0.001	0.917	0.344
AA homozygotes	5.71 ± 7.71	0.24 ± 0.75			
G allele carriers	4.58 ± 3.98	1.14 ± 2.38			
**Loneliness score** ^b^			< 0.001	0.970	0.088
AA homozygotes	45.43 ± 12.30	38.47 ± 10.21			
G allele carriers	49.75 ± 9.79	34.33 ± 7.33			

The data are presented as the means ± SD

^a^ One bipolar patient with G allele and 1 control with G allele did not complete the 17-item Hamilton Depression Rating Scale (HDRS) and were excluded from this calculation

^b^ One bipolar patient with G allele did not complete the loneliness and was excluded from this calculation

The BD patients in this study exhibited mean scores of 1.59 ± 3.14 on the YMRS and 5.00 ± 5.57 on the HDRS, indicating that a majority of the patients (66.7%) were in a euthymic state (scores of less than 7 on the HDRS and YMRS). For the distribution of the HDRS and YMRS scores, please see Supplementary Fig. 1. Compared to HC, BD patients demonstrated an elevated plasma OXT level and higher scores in mood symptoms (YMRS and HDRS), childhood trauma, and loneliness. However, there was no significant between-genotype difference observed for either rs53576 (Supplementary Table S1) or rs2228485 (Table 1).

### Genetic analysis

No significant differences between the BD patients and the HC were detected for the *OXTR* rs53576 or rs2228485 polymorphism (*p* = 0.899/0.253, respectively), and the genotype distribution did not deviate from Hardy–Weinberg equilibrium in both the BD (*p* = 0.6795/0.6282) and HC (*p* = 0.6406/0.7031). AG heterozygotes and GG homozygotes were considered as a single genotype (“G allele carriers”) based on their similar clinical characteristics (Lucht et al. 2009; McQuaid et al. 2013) and the limited number of cases in GG homozygotes. Notably, there was a significant interaction between rs53576 and rs2228485 polymorphisms (*p* < 0.001).

### Neuroimaging studies

**Fig. 1:**
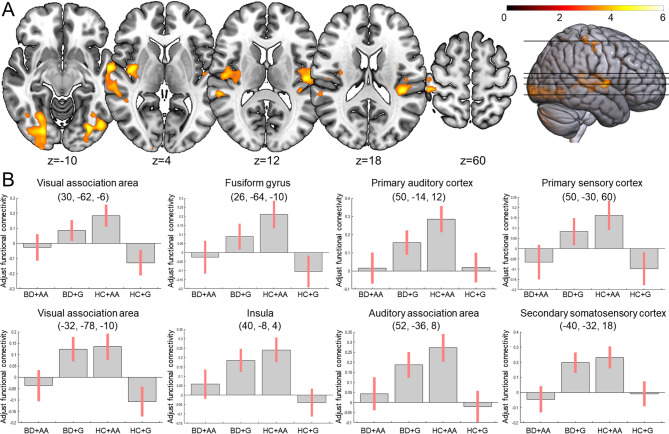
Regions showed significant functional connectivity with the left ventral striatum in the gene–disease interaction between the oxytocin receptor gene rs2228485 polymorphism and bipolar disorder. **(A)** There were gene–disease interactions in various brain regions, including the primary auditory cortex, auditory association area, visual association area, fusiform gyrus, primary somatosensory cortex, secondary somatosensory cortex, and insula. **(B)** AA homozygous healthy controls (HC + AA) exhibited higher functional connectivity compared to healthy controls with G allele (HC + G); however, this pattern reversed in bipolar disorder (BD) patients. Region coordinates are detailed in Supplementary Table S3. Similar results were observed in the right ventral striatum (see Supplementary Table S2). Results were thresholded at the uncorrected voxel level *p* = 0.001, followed by the cluster-level family-wise error rate (FWE)-corrected *p* = 0.05. The color bar represents the *t*-scores. Figures are displayed according to radiological convention (left = right)

Gene–disease interactions were observed in the primary auditory cortex, auditory association area, primary somatosensory cortex, and insula FC with the ventral striatum for both rs53576 (Supplementary Table S2) and rs2228485 (Fig. 1, Supplementary Table S3 and S4). While the results were similar between rs53576 and rs2228485, the findings were more robust for rs2228485, including additional ventral striatum FC with the visual association area, fusiform gyrus, and secondary somatosensory cortex. Therefore, subsequent correlation analyses focused exclusively on rs2228485.

Among HC, rs2228485 AA homozygotes exhibited increased ventral and dorsal striatocortical FC with the primary auditory cortex, auditory association area, and visual association area compared to G allele carriers (Supplementary Table S3, S4 and S5). Additionally, there were enhanced ventral striatocortical FC with the fusiform gyrus, primary somatosensory cortex, hippocampus, and amygdala (Supplementary Table S3 and S4). There was no significant difference between G allele carrier BD patients and AA homozygous BD patients.

In rs2228485 G allele carriers, BD patients demonstrated decreased ventral striatocortical FC with the primary auditory cortex and auditory association area compared to HC (Supplementary Table S3 and S4). Conversely, among rs2228485 AA homozygotes, BD patients exhibited increased ventral striatocortical FC with the visual association area and insula compared to HC (Supplementary Table S3 and S4).

### Correlation analysis between functional connectivity and the plasma Oxytocin level

Among HC, AA homozygotes exhibited a negative correlation between their plasma OXT level and dorsal striatocortical FC with the orbitofrontal cortex, meaning that lower OXT levels were associated with increased FC (Table 2 and Supplementary Fig. 2A). Conversely, BD patients with G allele showed positive correlations between their plasma OXT level and ventral or dorsal striatocortical FC with the posterior parietal cortex, meaning that higher OXT levels were associated with increased FC (Table 2, Supplementary Fig. 2B and 2 C). In contrast, no correlation was found in the HC with G allele or AA homozygous BD patients.

**Table 2:** Functional connectivity of ventral and dorsal striatum co-varying with the level of plasma Oxytocin

									Peak coordinate		
Seed	Lateral	Group	Genotype	Direction	Region	BA	Cluster	*Z* score	*x*	*y*	*z*
Dorsal striatum	Right	Controls	AA homozygotes	Negative	Orbitofrontal cortex	11	191	3.50	14	20	-12
		BD patients	G allele carriers	Positive	Posterior parietal cortex	39	169	4.07	-34	-58	36
Ventral striatum	Right	BD patients	G allele carriers	Positive	Posterior parietal cortex	39	237	3.77	-28	-68	44

Peak coordinates refer to the Montreal Neurological Institute (MNI) space. Significance was thresholded at the uncorrected voxel level *p* = 0.001, followed by the FWE-corrected cluster level *p* = 0.05. No correlation was found in AA homozygous BD patients or controls with G allele. BA, Brodmann area; BD, bipolar disorder

### Correlation analysis between functional connectivity and childhood trauma

Among HC, AA homozygotes displayed a positive correlation between their childhood trauma score and ventral striatocortical FC with the sensory association area, meaning that increased FC were associated with more childhood trauma reported (Table 3 and Supplementary Fig. 3A). In contrast, HC with G allele carriers exhibited negative correlations with the precuneus, meaning that decreased FC were associated with more childhood trauma reported (Table 3). On the other hand, BD patients with G allele showed negative correlations between their childhood trauma score and ventral striatocortical FC with the visual association area and posterior parietal cortex, meaning that decreased FC were associated with more childhood trauma reported (Table 3, Supplementary Fig. 3B and 3 C).

**Table 3:** Functional connectivity of ventral striatum co-varying with childhood trauma

									Peak coordinate		
Seed	Lateral	Group	Genotype	Direction	Region	BA	Cluster	*Z* score	*x*	*y*	*z*
Ventral striatum	Left	Controls	AA homozygotes	Positive	Sensory association area	5	128	3.21	6	-46	76
			G allele carriers	Negative	Precuneus	7	160	5.14	6	-56	60
					Precuneus	7	135	4.30	-4	-70	42
	Left	BD patients	G allele carriers	Negative	Visual association area	18	186	4.05	30	-90	-8
					Visual association area	19	—	3.56	44	-86	-12
					Posterior parietal cortex	39	199	4.57	38	-62	28
					Posterior parietal cortex	39	132	4.17	-38	-68	34
					Posterior parietal cortex	7	129	3.57	-36	-46	62
	Right	BD patients	G allele carriers	Negative	Visual association area	19	136	4.36	-36	-76	-18

Peak coordinates refer to the Montreal Neurological Institute (MNI) space. Significance was thresholded at the uncorrected voxel level *p* = 0.001, followed by the FWE-corrected cluster level *p* = 0.05. No correlation was found in the dorsal striatum. BA, Brodmann area; BD, bipolar disorder

### Sensitivity analysis

The results of these analyses revealed that while the cluster size of significant brain regions was reduced, the primary findings concerning the key striatocortical connectivity patterns remained largely consistent and significant (Supplementary Table S6). This suggests that our main conclusions are robust and not influence by the inclusion of non-euthymic patients.

## Discussion

To the best of our knowledge, this is the first study to elucidate the underlying neurocircuitry of OXT bi-direction effects in a gene–disease interaction model involving rs53576 (Supplementary Table S2) and rs2228485 (Fig. 1, Supplementary Table S3, S4 and S5). The gene–disease interaction was more robust in rs2228485, with the AA homozygotes exhibiting enhanced striatocortical FC to the sensory association areas and limbic areas in HC, which was correlated with their plasma OXT level (Table 2). In contrast, the alterations in ventral striatocortical FC are reversed among BD patients, who had relative more childhood trauma (i.e., negative environments), showing hypo-FC in rs2228485 AA homozygotes but hyper-FC in rs2228485 G allele carriers (Supplementary Table S3 and S4). This opposing pattern of connectivity differences between groups depending on their genotype is the very definition of a gene–disease interaction. Furthermore, we found oxytocinergic modulation of the attention networks (Table 2, Supplementary Fig. 2B and 2 C) that were influenced by childhood trauma (Table 3 and Supplementary Fig. 3C).

The rs2228485 AA homozygous HC, in comparison to HC with G allele, exhibited enhanced ventral striatocortical FC with three unimodal sensory association areas (i.e., auditory, visual and somatosensory; see Fig. 1, Supplementary Table S3 and S4), indicating a better ability in sensory processing and recognition. The regulation of sensory processing by OXT is well-documented; there are *OXTR* expressions within and projections of oxytocinergic neurons axons to the primary auditory cortex, primary visual cortex and somatosensory cortex (Grinevich and Stoop 2018). Furthermore, OXT is involved in multiple dimensions of maternal behaviors, including mother-to-infant cries (Schiavo et al. 2020) and mother-to-infant gaze (Kim et al. 2014), while *OXTR* SNP may influence sensitivity in male-to-female cries (Truzzi et al. 2018). In a way, these social behaviors are a kind of neurobehavioral responses modulated by OXT (Grinevich and Stoop 2018). As mentioned earlier, *OXTR* rs2228485 polymorphism is associated with a better ability to read emotional responses in male faces (Lucht et al. 2013), and herein, our data provide a possible underlying neurocircuitry via the ventral striatocortical pathway.

Following the sensory areas, in rs2228485 AA homozygous HC, we observed enhanced ventral striatocortical FC with the amygdala, fusiform gyrus and hippocampus (Supplementary Table S3 and S4). These regions are all involved in recapitulating emotional context (Fenker et al. 2005). The amygdala assigns emotional value to sensory stimuli (Sah et al. 2003), encodes emotional value with the fusiform gyrus (Petrovic et al. 2008), and passes sensory information to the hippocampus, where it encodes emotional memories (Abramova et al. 2020; Richardson et al. 2004). OXT is known to be involved in the formation of social and emotional memory (Abramova et al. 2020), and the *OXTR* is expressed in the amygdala (Aspé-Sánchez et al. 2015) and hippocampus (Abramova et al. 2020). Furthermore, OXT is known to affect the excitation/inhibition balance of neuronal circuits that open and close sensitivity to external inputs in the hippocampus (Grinevich and Stoop 2018).

Taken together, our results in HC support a model in which the *OXTR* rs2228485 polymorphism influences the striatocortical FC with the sensory association areas and limbic areas (Fig. 1, Supplementary Table S3 and S4). Our reasoning is corroborated by the correlation between the plasma OXT level and the striatocortical FC with the orbitofrontal cortex in AA homozygous HC (Table 2 and Supplementary Fig. 2A). The orbitofrontal cortex, a part of the limbic area (Catani et al. 2013), is modulated by OXT (Xu et al. 2019), while both the orbitofrontal cortex and striatum lie in the route where OXT is delivered (Lee et al. 2020).

Although enhanced FC may offer sufficient accuracy and sensitivity for detecting social context in rs2228485 AA homozygous HC (Grinevich and Stoop 2018), such sensitivity may also be vulnerable to negative environments (Tabak 2013), including childhood trauma. Our rationale is supported by a positive correlation between childhood trauma and ventral striatocortical FC to the sensory association area in the rs2228485 AA homozygous HC; the higher the FC, the more the childhood trauma reported (Table 3 and Supplementary Fig. 3A). Moreover, the aforementioned ventral striatocortical FC changes are reversed among BD patients, who had more childhood trauma, with hypo-FC in rs2228485 AA homozygotes but hyper-FC in rs2228485 G allele carriers (Supplementary Table S3 and S4). Collectively, subjects with “sensitive” allele (i.e., AA homozygotes) may ultimately exhibit increased or decreased FC depending upon positive or negative environments (Fig. 1).

In our previous study, we reported that the striatocortical FC to the posterior parietal cortex is a critical circuitry in the oxytocinergic modulation of the reward system (Wei et al. 2020). In line with this, we found such FC positively correlated with the plasma OXT level in BD patients with the rs2228485 G allele (Table 2, Supplementary Fig. 2B and 2 C), indicating OXT-driven modulation in attention networks. OXT increases the FC between striatocortical circuitry (Bethlehem et al. 2017), and the posterior parietal cortex is engaged in both top-down and bottom-up attention (Katsuki and Constantinidis 2014), starting with visual cortical pathways and branching into the ventral pathway (via fusiform gyrus) and the dorsal pathway (via posterior parietal cortex and frontal eye field).

Furthermore, the striatocortical FC to the posterior parietal cortex is also correlated with the childhood trauma in BD patients with the rs2228485 G allele (Table 3 and Supplementary Fig. 3C), implying an interaction between negative environments and attention networks. Therefore, although the rs2228485 G allele seems to be a protective polymorphism in BD patients, childhood trauma still has negative effects on the striatocortical FC in BD patients with G allele (Table 3). As impaired function of OXT is associated with the development of neuropsychiatric disorders (Abramova et al. 2020; Meyer-Lindenberg and Tost 2012), cumulative negative effects on the striatocortical FC may ultimately increase vulnerability to BD.

Therefore, healthy adolescents or young adults carrying the G-allele who have a documented history of significant childhood adversity may represent a vulnerable subgroup. These individuals could benefit from early intervention programs aimed at enhancing resilience and mitigating the neurobiological effects of early life stress before the full onset of BD. In addition, intranasal oxytocin is currently being investigated as a potential treatment for various psychiatric conditions (Quintana et al. 2017). Our findings suggest that treatment efficacy may be moderated by *OXTR* genotype. Future clinical trials could incorporate genetic profiling to predict treatment response or guide personalized interventions for optimal therapeutic outcomes.

In this study, we investigated the interactions between *OXTR* SNPs and BD in relation to striatocortical FC as a key neural substrate, with childhood trauma considered as a potential stressor. Although no significant between-group differences in *OXTR* SNPs were observed, assessing the direct association between *OXTR* SNPs and BD was not the primary objective. Instead, the rs53576 and rs2228485 genotypes were utilized solely for grouping purposes. It is important to recognize that disease vulnerability is influenced not only by a single SNP but also by the interactions and cumulative effects of multiple genes. While significant between-genotype differences and interactions were observed in FC, no such differences were found in childhood trauma, depression, or loneliness scores. *FC* may offer greater sensitivity than behavioral assessments, as they can capture subclinical or preclinical alterations. This distinction arises because genetic effects may exhibit stronger penetrance at the neurobiological level compared to overt behavioral manifestations (Bigos and Weinberger 2010; Rasetti and Weinberger 2011; Tan et al. 2008).

Our data provide a possible underlining neurocircuitry for the *OXTR* rs2228485 polymorphism on the ventral and dorsal striatocortical circuitry in HC and BD patients, including sensory association areas and limbic areas. Furthermore, we found oxytocinergic modulation of the attention networks that were influenced by childhood trauma. Our data provide a possible explanation for better sensory recognition and less negative emotion in the rs2228485 polymorphism in HC. Our preliminary report invites future studies of a larger sample size for verification and to further explore whether such interactions can be generalized to other *OXTR* polymorphisms.

### Limitations

Our study had certain limitations. As a cross-sectional study, establishing conclusive evidence for consequential or causal roles among OXT, childhood trauma, and striatocortical connectivity is challenging. Nonetheless, we present a model that incorporates *OXTR* SNP (innate mechanisms) and concurrent environments during critical developmental periods (acquired mechanisms) to elucidate the possible underlying neurocircuitry of gene–disease interactions. Another concern is the lack of a reliable OXT assay, which is a well-known issue in this field (Poljak and Sachdev 2021). OXT immunoreactivity levels cannot accurately infer true values of OXT, and therefore cannot be compared between studies (Leng and Sabatier 2016; McCullough et al. 2013; Robinson et al. 2014). However, measuring absolute values of OXT in the subjects is not the primary objective of our current imaging genetics study. Instead, we used these assays to compare relative levels of peripheral OXT between genotypes and groups (Lawson 2017), as well as to investigate the relationship between OXT level and FC. Third, while most patients were euthymic, approximately 35% were not. The inclusion of patients in different mood states could have introduced heterogeneity and potentially influenced the results. Nevertheless, we conducted a sensitivity analysis including only euthymic BD patients, and the key striatocortical connectivity patterns remained largely consistent and significant (Supplementary Table S6). This suggests that our main conclusions are robust and were not significantly influenced by the inclusion of non-euthymic patients. Forth, we acknowledge that psychotropic medications could be a potential confounder. Lithium is known to exert broad neurobiological effects, including modulation of functional brain connectivity (Bergamelli et al. 2021). While direct evidence for the effects of antipsychotics on functional connectivity in BD is limited, such effects have been demonstrated in schizophrenia using longitudinal fMRI studies (Abbott et al. 2013). Additionally, antipsychotics have been shown to influence oxytocinergic neurons in animal models (Kiss and Osacka 2021). Given the clinical reality of treating BD, most patients were on necessary and heterogeneous medication regimens. As such, it is difficult to isolate the specific effects of medications from the underlying disease processes in the current design. Fifth, the limited sample size may have reduced statistical power and increased the risk of false negatives (Type II errors). Future studies involving larger, medication-naive or stratified samples would be essential to clarify these potential confounds.

## Supplementary Information

Below is the link to the electronic supplementary material.

Supplementary Material 1

## Data Availability

The data used to support the findings of this study are available from the corresponding author upon request.
