# Supplementary material for: Pleiotropic effects of oxytocin receptor polymorphisms: influencing striatocortical connectivity in bipolar disorder
Source: Int J Bipolar Disord. 2025 Aug 31;13:25. doi: 10.1186/s40345-025-00393-8 (PMC12399500; doi:10.1186/s40345-025-00393-8)
Supplement: Supplementary file 1 — Supplementary Material 1 [file 40345_2025_393_MOESM1_ESM.docx]

**Supplementary Methods**

Since the Hamilton Depression Rating Scale (HDRS) score for healthy controls (HC) was low (0.65 ± 1.723), correlation analyses were not conducted in this group. In the bipolar disorder (BD) group, one-tailed two-sample *t*-tests were used to examine the correlation between HDRS scores and functional connectivity (FC) for each genotype. Additionally, for both HC and BD groups, one-tailed two-sample *t*-tests were performed to assess the correlation between FC and loneliness scores for each genotype. Demeaned values (in SPM) were entered as regressors to identify brain regions exhibiting positive or negative correlations with FC in each genotype within the BD and HC groups. Statistical significance was set at an uncorrected voxel-level threshold of *p* = 0.001, followed by a family-wise error (FWE)-corrected cluster-level threshold of *p* = 0.05. To visualize the regression results, scatterplots were generated using FC values extracted from brain regions within a 3-mm radius of the peak MNI coordinates (listed in Table 2, Table 3, Supplementary Table S7, and Supplementary Table S8). The corresponding correlation coefficients (*r*) and *p*-values were analyzed using SPSS Statistics 20.0.

**Supplementary Data**

***Correlation analysis between functional connectivity and the depression***

Among BD patients, AA homozygotes exhibited positive correlations between their depression score and ventral striatocortical FC with primary auditory cortex and auditory association area (Supplementary Table S7 and Supplementary Fig.4A). In contrast, the BD patients with G allele showed negative correlations between their depression score and dorsal striatocortical FC with visual association area, fusiform gyrus, primary somatosensory cortex, and primary motor cortex (Supplementary Table S7, Supplementary Fig.4B and 4C).

***Correlation analysis between functional connectivity and the loneliness***

Among HC, AA homozygotes showed a negative correlation between their loneliness score and ventral striatocortical FC with posterior parietal lobe (Supplementary Table S8 and Supplementary Fig.5A). Similarly, the BD patients with G allele showed negative correlations between their loneliness score and ventral striatocortical FC with posterior parietal lobe and premotor cortex (Supplementary Table S8 and Supplementary Fig.5B), as well as dorsal striatocortical FC with frontal eye fields (Supplementary Table S8). In contrast, The AA homozygous BD patients exhibited a negative correlation between their loneliness score and ventral striatocortical FC with anterior cingulate cortex (Supplementary Table S8 and Supplementary Fig.5C). No correlation was found in the HC with G allele.

**Supplementary Discussion**

The rs2228485 AA homozygous BD patients exhibited ventral striatocortical hypo-FC with primary auditory cortex and auditory association area that were positively correlated with their depression score (Supplementary Table S7 and Supplementary Fig.4A), suggesting an adaptive FC alteration to auditory salience; the lower the FC, the lower the depression. Nevertheless, the rs2228485 AA homozygous BD patients, with relatively higher plasma OXT level, remain subclinical high in the depression score (Table 1). On the other hand, the BD patients with rs2228485 G allele showed ventral striatocortical hyper-FC with visual association area that was negatively correlated with their depression score (Supplementary Table S7 and Supplementary Fig.4B), connoting different adaptive neuroplasticity.

Moreover, only among the rs2228485 AA homozygous HC and the BD patients with rs2228485 G allele were the loneliness negatively correlated with the striatocortical FC to the posterior parietal cortex (Supplementary Table S8, Supplementary Fig.5A and 5B), suggesting an adaptive emotional regulation by attention networks. This may explain how does the rs2228485 polymorphism be associated with lower score for loneliness in HC [1] , and extend possible effects in BD patients. Interestingly, the BD patients with rs2228485 G allele also exhibit negative correlations between the loneliness and the ventral or dorsal striatocortical FC to the premotor cortex or frontal eye field, respectively (Supplementary Table S8). Given that the OXT-driven modulation may influence social behaviors through the control of the motor activity in prior animal studies [2,3], our study provides mechanistic insights via the striatocortical circuitry in human.

Collectively, the aforementioned dorsal and ventral striatocortical FC to the sensory association areas and attention networks were correlated with depression (Supplementary Table S7, Supplementary Fig.4A and 4B) and loneliness (Supplementary Table S8, Supplementary Fig.5A and 5B), respectively. The differential changes among BD patients may indicate different adaptive neuroplasticity in BD patients with different polymorphisms.

**References**

1. Lucht MJ, Barnow S, Sonnenfeld C, Rosenberger A, Grabe HJ, Schroeder W *et al.* Associations between the oxytocin receptor gene (OXTR) and affect, loneliness and intelligence in normal subjects. *Progress in neuro-psychopharmacology & biological psychiatry* 2009; **33**(5)**:** 860-866.

2. Patel JC, Rossignol E, Rice ME, Machold RP. Opposing regulation of dopaminergic activity and exploratory motor behavior by forebrain and brainstem cholinergic circuits. *Nature communications* 2012; **3:** 1172.

3. Charlet A, Grinevich V. Oxytocin Mobilizes Midbrain Dopamine toward Sociality. *Neuron* 2017; **95**(2)**:** 235-237.

**Supplementary Fig. 1.** The distribution of the **(****A)** Young Mania Rating Scale and the **(B)** Hamilton Depression Rating Scale scores.

A


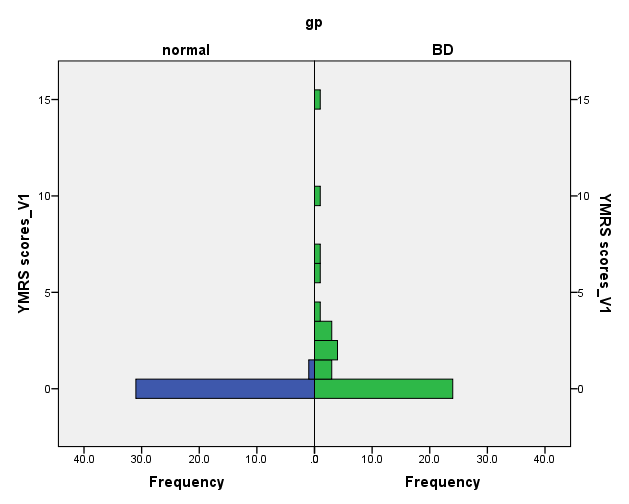


B


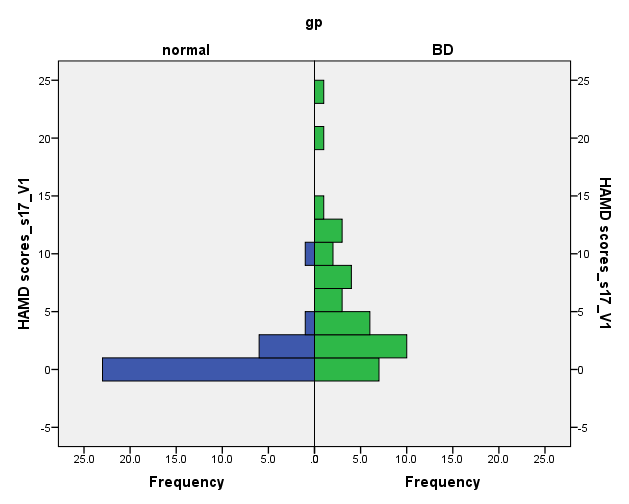


**Supplementary Fig. 2.** Ventral and dorsal striatal functional connectivity covaries with the level of plasma oxytocin.


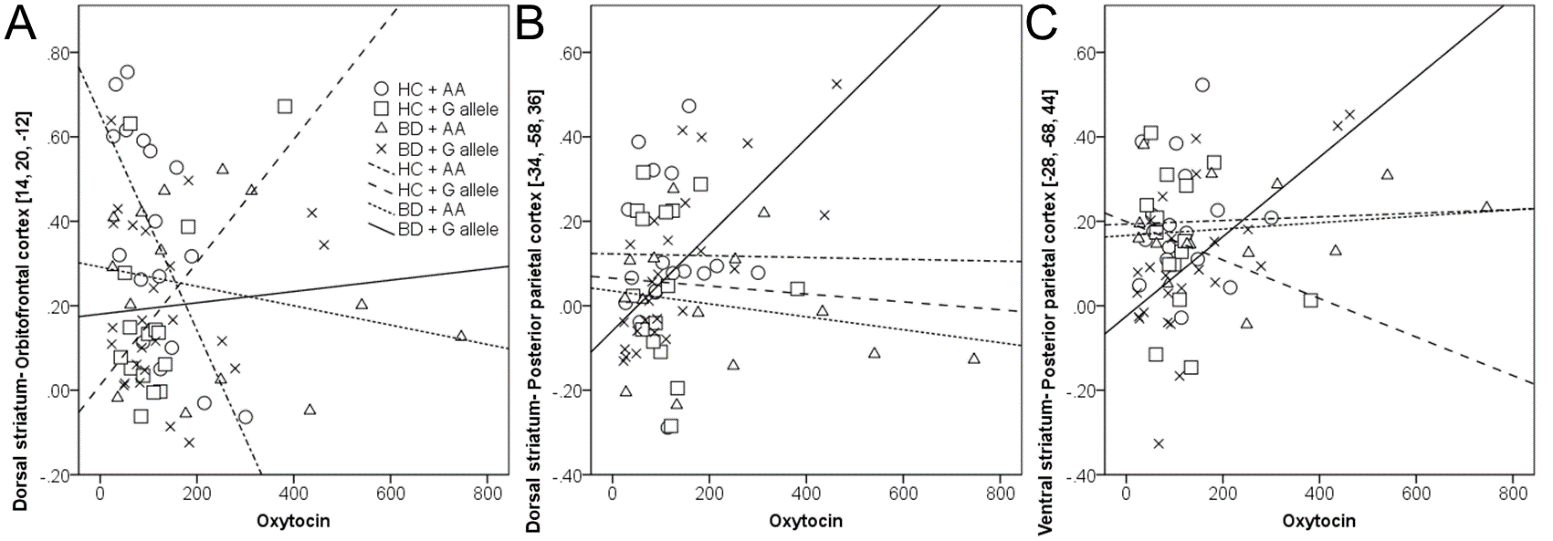


The level of plasma oxytocin was negatively correlated with the functional connectivity between the dorsal striatum and the **(A)** orbitofrontal cortex (*r* = -0.697, *p* = 0.002) in the AA homozygous healthy controls (HC), but this was not the case in other subgroups (HC with G allele: *r* = 0.540, *p* = 0.038, not significant after the correction for multiple comparisons in SPM; AA homozygous bipolar disorder [BD] patients: *r* = -0.239, *p* = 0.411; BD patients with G allele: *r* = 0.081, *p* = 0.701). In contrast, the level of plasma oxytocin was positively correlated with the functional connectivity between the **(B)** dorsal or **(C)** ventral striatum to the posterior parietal cortex (*r* = 0.711/0.619, *p* = 0.000/0.001) in the BD patients with G allele, but this was not the case in other subgroups (AA homozygous HC: *r* = -0.009/0.022, *p* = 0.974/0.932; HC with G allele: *r* = -0.042/-0.235, *p* = 0.882/0.399; AA homozygous BD patients: *r* = -0.214/0.146, *p* = 0.462/0.619, respectively). The scatterplots disclose the relationship between the level of plasma oxytocin and the ventral or dorsal striatal functional connectivity around peak voxel (see Table 2). Significance was thresholded at the uncorrected voxel level *p* = 0.001, followed by the FWE-corrected cluster level *p* = 0.05.

**Supplementary Fig. 3.** Ventral striatal functional connectivity covaries with childhood trauma score.


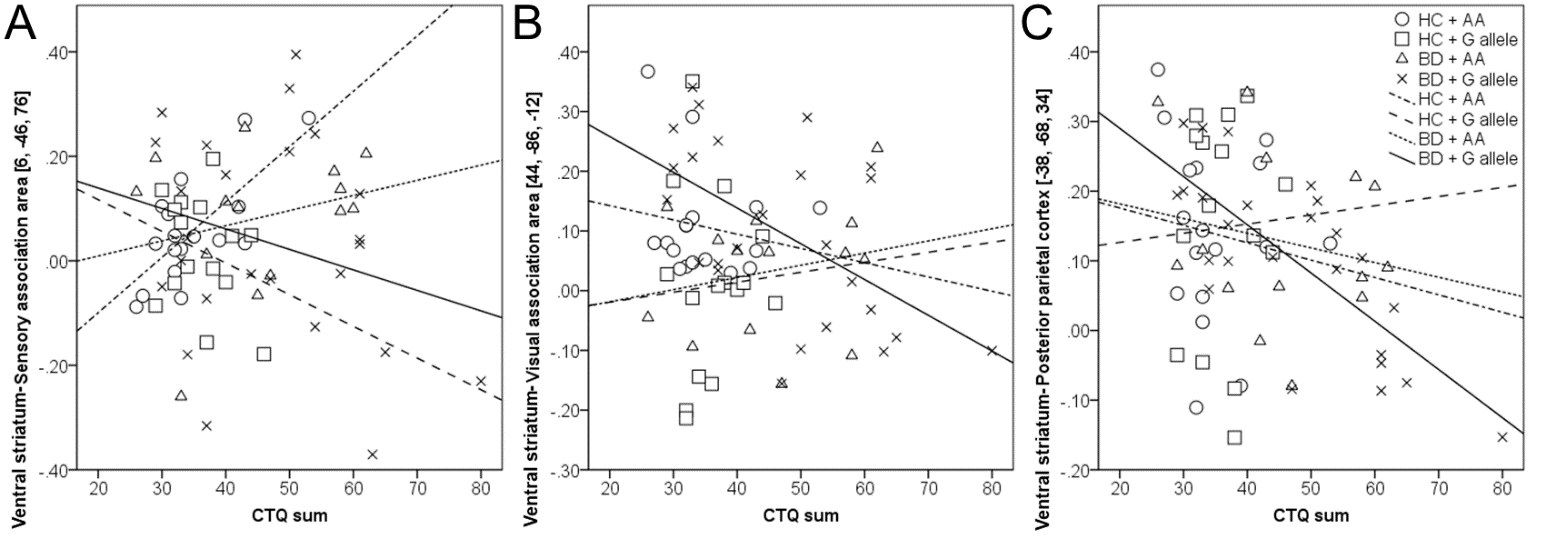


The Childhood Trauma Questionnaire (CTQ) score was positively correlated with the functional connectivity between the ventral striatum and the **(A)** sensory association area (*r* = 0.716, *p* = 0.001) in the AA homozygous healthy controls (HC), but this was not the case in other subgroups (HC with G allele: *r* = -0.283, *p* = 0.306; AA homozygous bipolar disorder [BD] patients: *r* = 0.261, *p* = 0.367; BD patients with G allele: *r* = -0.268, *p* = 0.196). In contrast, the CTQ score was negatively correlated with the functional connectivity between the ventral striatum to the **(B)** visual association area (*r* = -0.553, *p* = 0.004) and **(C)** posterior parietal cortex (*r* = -0.754, *p* < 0.001) in the BD patients with G allele, but this was not the case in other subgroups (AA homozygous HC: *r* = -0.181/-0.133, *p* = 0.486/0.610; HC with G allele: *r* = 0.055/0.042, *p* = 0.846/0.883; AA homozygous BD patients: *r* = 0.219/-0.204, *p* = 0.451/0.485, respectively). The scatterplots disclose the relationship between the CTQ score and the ventral striatal functional connectivity around peak voxel (see Table 3). Significance was thresholded at the uncorrected voxel level *p* = 0.001, followed by the FWE-corrected cluster level *p* = 0.05.

**Supplementary Fig. 4.** Ventral and dorsal striatal functional connectivity covaries with depression score.


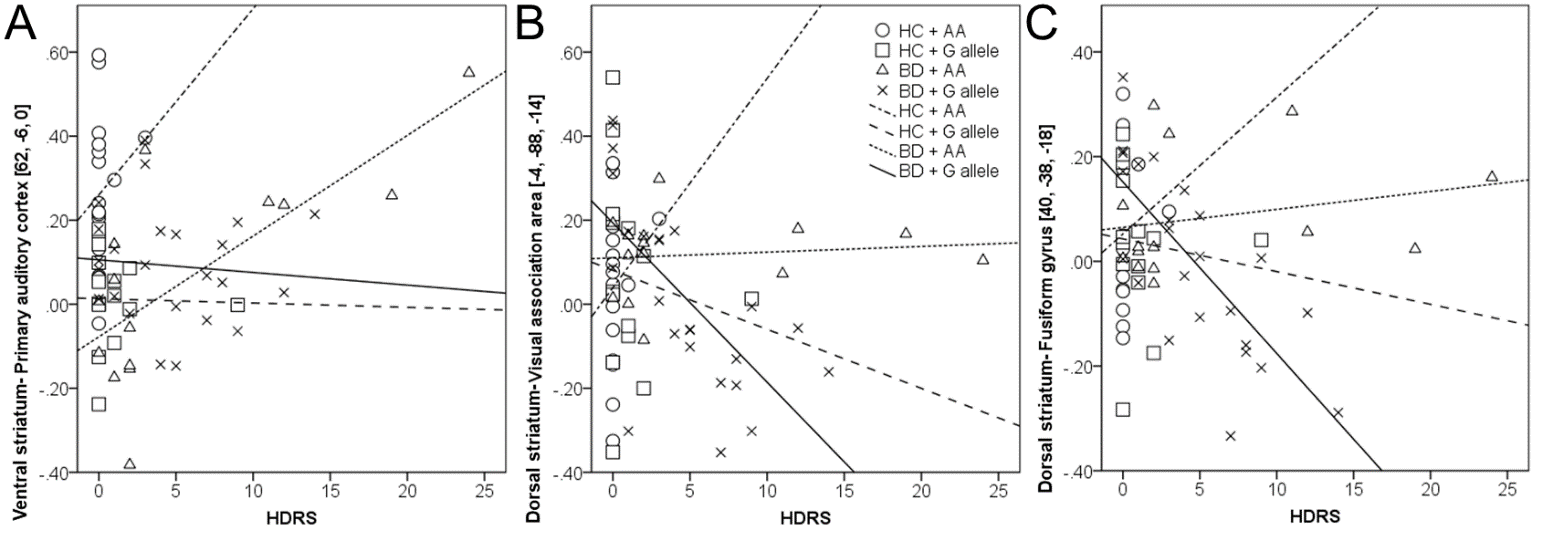


The Hamilton Depression Rating Scale (HDRS) score was positively correlated with the functional connectivity between the ventral striatum and the **(A)** primary auditory cortex (*r* = 0.731, *p* = 0.003) in the AA homozygous bipolar disorder (BD) patients, but this was not the case in other subgroups (AA homozygous healthy controls [HC]: *r* = 0.192, *p* = 0.461; HC with G allele: *r* = -0.022, *p* = 0.940; BD patients with G allele: *r* = -0.089, *p* = 0.679, respectively). In contrast, the HDRS score was negatively correlated with the functional connectivity between the dorsal striatum to the **(B)** visual association area (*r* = -0.664, *p <* 0.001) and **(C)** fusiform gyrus in the BD patients with G allele (*r* = -0.748, *p <* 0.001), but this was not the case in other subgroups (AA homozygous HC: *r* = 0.203/0.141, *p* = 0.435/0.591; HC with G allele: *r* = -0.141/-0.104, *p* = 0.629/0.723; AA homozygous BD patients: *r* = 0.110/0.229, *p* = 0.707/0.432). The scatterplots disclose the relationship between the depression score and the ventral or dorsal striatal functional connectivity around peak voxel (see Supplementary Table S7). Significance was thresholded at the uncorrected voxel level *p* = 0.001, followed by the FWE-corrected cluster level *p* = 0.05.

**Supplementary Fig. 5.** Ventral striatal functional connectivity covaries with loneliness score.


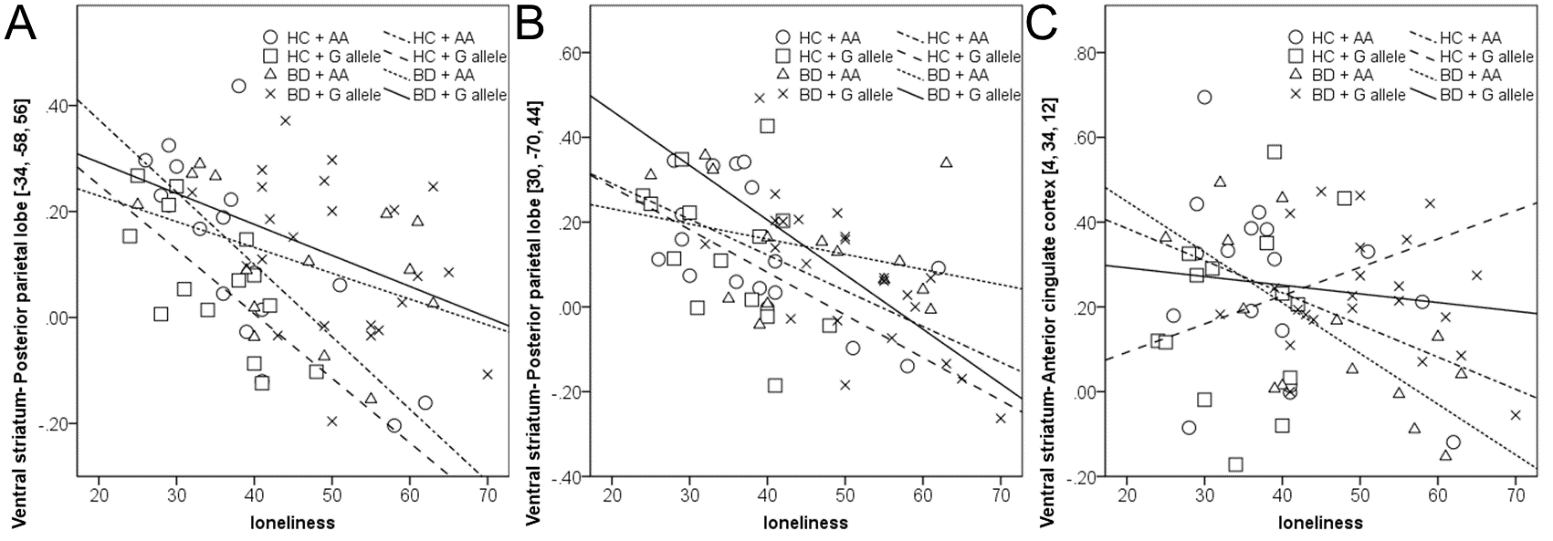


The loneliness score was negatively correlated with the functional connectivity between the ventral striatum and the posterior parietal lobe in **(A)** AA homozygous HC (*r* = -0.771, *p* < 0.001) and **(B)** G allele BD patients (*r* = -0.719, *p* < 0.001), but this was not the case in other subgroups (G allele HC: *r* = -0.698/-0.433, *p* = 0.005/0.122, not significant after the correction for multiple comparisons in SPM; AA homozygous BD patients: *r* = -0.435/-0.315, *p* = 0.120/0.272, respectively). The loneliness score was negatively correlated with the functional connectivity between the ventral striatum and the **(C)** anterior cingulate cortex in AA homozygous BD patients (*r* = -0.725, *p* = 0.003), but this was not the case in other subgroups (AA homozygous HC: *r* = -0.367, *p* = 0.147; G allele HC: *r* = 0.233, *p* = 0.423; G allele BD patients: *r* = -0.139, *p* = 0.528). The scatterplots disclose the relationship between the loneliness score and the ventral striatal functional connectivity around the peak voxel (see Supplementary Table S8). Significance was thresholded at the uncorrected voxel level *p* = 0.001, followed by the FWE-corrected cluster level *p* = 0.05.

**Supplementary Table S1.**

Demographic data and baseline information between the oxytocin receptor gene rs53576 polymorphism and bipolar disorder.

|  | Bipolar patients (*n*=39) | Controls (*n*=32) |  | *p* Value |  |
| --- | --- | --- | --- | --- | --- |
|  |  |  | Group | Genotype | Interaction |
| **Age, year** |  |  | 0.075 | 0.625 | 0.092 |
| AA homozygotes | 41.05±14.48 | 31.29±8.53 |  |  |  |
| G allele carriers | 34.95±12.18 | 34.67±9.20 |  |  |  |
| **Gender, female** **(%)** |  |  | 0.259 | 0.303 |  |
| AA homozygotes | 15 (79%) | 8 (57%) | 0.178 |  |  |
| G allele carriers | 12 (60%) | 10 (56%) | 0.782 |  |  |
| **Plasma oxytocin (pg/ml)** |  |  | 0.092 | 0.747 | 0.377 |
| AA homozygotes | 175.58±187.88 | 92.83±44.44 |  |  |  |
| G allele carriers | 157.56±141.88 | 131.51±91.11 |  |  |  |
| **Childhood trauma** |  |  | <0.001 | 0.791 | 0.816 |
| AA homozygotes | 46.37±11.43 | 34.79±7.32 |  |  |  |
| G allele carriers | 46.45±14.56 | 36.06±5.06 |  |  |  |
| **YMRS score** |  |  | 0.007 | 0.557 | 0.644 |
| AA homozygotes | 1.89±4.00 | 0.07±0.27 |  |  |  |
| G allele carriers | 1.30±2.08 | 0.00±0.00 |  |  |  |
| **HDRS score**^a^ |  |  | <0.001 | 0.320 | 0.156 |
| AA homozygotes | 6.26±6.91 | 0.38±0.87 |  |  |  |
| G allele carriers | 3.74±3.56 | 0.83±2.15 |  |  |  |
| **Loneliness score**^b^ |  |  | <0.001 | 0.393 | 0.797 |
| AA homozygotes | 48.89±10.68 | 38.07±11.67 |  |  |  |
| G allele carriers | 47.42±11.21 | 35.33±6.54 |  |  |  |

The data are presented as the means ± SD.

^a^ One bipolar patient with G allele and 1 AA homozygous control did not complete the 17-item Hamilton Depression Rating Scale (HDRS) and were excluded from this calculation.

^b^ One bipolar patient with G allele did not complete the loneliness and was excluded from this calculation.

**Supplementary Table S2.**

Peak MNI coordinates of the regions exhibiting significant resting-state functional connectivity with the right ventral striatum with between-rs53576 genotype and between-group differences

|  |  |  |  |  | Peak coordinate | | |
| --- | --- | --- | --- | --- | --- | --- | --- |
| Contrast | Region | BA | Cluster | *Z* score | *x* | *y* | *z* |
| (AA homozygotes > G allele carriers) (controls > BD patients) | Primary auditory cortex | 41 | 179 | 4.17 | 40 | -20 | 12 |
|  | Insula | 13 | — | 3.91 | 38 | -16 | 0 |
|  | Auditory association area | 22 | — | 3.88 | 44 | -16 | -4 |
|  | Primary somatosensory cortex | 1 | 172 | 3.73 | -48 | -16 | 10 |
|  | Premotor cortex | 6 | — | 3.71 | -48 | -10 | 6 |
|  | Ventrolateral prefrontal cortex | 44 | — | 3.52 | -58 | 4 | 4 |
| AA homozygous BD patients < AA homozygous controls | Primary auditory cortex | 41 | 171 | 3.82 | -48 | -18 | 10 |
|  | Secondary somatosensory cortex | 40 | — | 3.34 | -46 | -28 | 14 |
| G allele carrier BD patients > G allele carrier controls | Visual association area | 18 | 158 | 3.90 | 14 | -90 | 20 |

Peak coordinates refer to the Montreal Neurological Institute space. Significance was thresholded at the uncorrected voxel level *p*=0.001, followed by the FWE-corrected cluster level *p*=0.05. BA, Brodmann area; BD, bipolar disorder.

**Supplementary Table S3.**

Peak MNI coordinates of the regions exhibiting significant resting-state functional connectivity with the left ventral striatum with between-rs2228485 genotype and between-group differences

|  |  |  |  |  | Peak coordinate | | |
| --- | --- | --- | --- | --- | --- | --- | --- |
| Contrast | Region | BA | Cluster | *Z* score | *x* | *y* | *z* |
| (AA homozygotes > G allele carriers) (controls > BD patients) | Visual association area | 19 | 705 | 4.86 | -32 | -78 | -10 |
|  | Visual association area | 19 | 993 | 4.12 | 30 | -62 | -6 |
|  | Fusiform gyrus | 37 | — | 4.06 | 26 | -64 | -10 |
|  | Secondary somatosensory cortex | 40 | 543 | 4.67 | -40 | -32 | 18 |
|  | Primary auditory cortex | 41 | — | 3.85 | -48 | -12 | 8 |
|  | Auditory association area | 22 | — | 3.57 | -60 | -28 | 6 |
|  | Primary somatosensory cortex | 1 | 159 | 4.16 | 50 | -30 | 60 |
|  | Auditory association area | 22 | 526 | 4.49 | 52 | -36 | 8 |
|  | Insula | 13 | 480 | 4.40 | 40 | -8 | 4 |
|  | Primary auditory cortex | 41 | — | 4.07 | 50 | -14 | 12 |
| AA homozygous controls > G allele carrier controls | Primary auditory cortex | 41 | 1638 | 4.95 | 68 | -6 | 2 |
|  | Auditory association area | 22 | — | 4.37 | 50 | -38 | 10 |
|  | Primary auditory cortex | 41 | 480 | 4.34 | -46 | -12 | 8 |
|  | Secondary somatosensory cortex | 40 | — | 4.19 | -56 | -24 | 10 |
|  | Visual association area | 19 | 1526 | 4.77 | 24 | -80 | -10 |
|  | Fusiform gyrus | 37 | — | 4.54 | 28 | -66 | -12 |
|  | Visual association area | 19 | 635 | 4.37 | -48 | -74 | -10 |
|  | Hippocampus | — | 213 | 4.53 | 28 | -16 | -16 |
|  | Amygdala | — | — | 4.03 | 26 | -2 | -18 |
|  | Amygdala | — | 213 | 4.17 | -24 | -4 | -20 |
|  | Primary somatosensory cortex | 1 | 750 | 4.06 | 46 | -30 | 58 |
| AA homozygous BD patients < AA homozygous controls | Primary auditory cortex | 41 | 256 | 4.10 | 64 | -6 | 4 |
|  | Auditory association area | 22 | — | 3.64 | 68 | -18 | 4 |
|  | Primary auditory cortex | 41 | 145 | 3.99 | -48 | -18 | 10 |
|  | Secondary somatosensory cortex | 40 | 154 | 3.85 | -42 | -32 | 18 |
|  | Corpus callosum | — | 150 | 4.20 | 10 | 12 | 20 |
| G allele carrier BD patients > G allele carrier controls | Insula | 13 | 249 | 4.25 | 36 | 20 | -4 |
|  | Insula | 13 | 141 | 3.77 | 38 | -6 | 4 |
|  | Visual association area | 19 | 209 | 4.19 | -48 | -74 | -10 |
|  | Visual association area | 19 | 176 | 3.57 | -30 | -50 | -4 |
|  | Visual association area | 18 | 173 | 3.68 | 22 | -80 | -4 |
| G allele carrier BD patients *vs*. AA homozygous BD patients | NA | NA | NS | NS | NA | NA | NA |

Peak coordinates refer to the Montreal Neurological Institute space. Significance was thresholded at the uncorrected voxel level *p*=0.001, followed by the FWE-corrected cluster level *p*=0.05. BA, Brodmann area; BD, bipolar disorder; NA, not available; NS, not significant.

**Supplementary Table S4.**

Peak MNI coordinates of the regions exhibiting significant resting-state functional connectivity with the right ventral striatum with between-rs2228485 genotype and between-group differences

|  |  |  |  |  | Peak coordinate | | |
| --- | --- | --- | --- | --- | --- | --- | --- |
| Contrast | Region | BA | Cluster | *Z* score | *x* | *y* | *z* |
| (AA homozygotes > G allele carriers) (controls > BD patients) | Primary auditory cortex | 41 | 500 | 4.48 | 38 | -20 | 12 |
|  | Primary somatosensory cortex | 1 | — | 4.48 | 48 | -14 | 12 |
|  | Insula | 13 | — | 4.30 | 42 | -8 | 4 |
|  | Auditory association area | 22 | 169 | 4.03 | 64 | -24 | 0 |
|  | Secondary somatosensory cortex | 40 | 387 | 3.73 | -42 | -28 | 16 |
|  | Primary auditory cortex | 41 | — | 3.68 | -58 | -28 | 12 |
|  | Auditory association area | 22 | — | 3.57 | -60 | -34 | 16 |
|  | Visual association area | 19 | 445 | 4.17 | -32 | -74 | -14 |
|  | Fusiform gyrus | 37 | 148 | 3.91 | 28 | -66 | -6 |
|  | Visual association area | 19 | — | 3.47 | 22 | -70 | -12 |
|  | Insula | 13 | 172 | 3.84 | -48 | 4 | -6 |
|  | Hippocampus | — | 197 | 3.54 | -24 | -8 | -20 |
| AA homozygous controls > G allele carrier controls | Primary auditory cortex | 41 | 1361 | 5.25 | 68 | -8 | 0 |
|  | Hippocampus | — | — | 4.93 | 28 | -14 | -16 |
|  | Primary auditory cortex | 41 | 526 | 4.87 | -46 | -12 | 8 |
|  | Auditory association area | 22 | — | 4.57 | -62 | -30 | 16 |
|  | Visual association area | 19 | 507 | 4.71 | -32 | -74 | -16 |
|  | Fusiform gyrus | 37 | — | 3.98 | -42 | -68 | -18 |
|  | Visual association area | 18 | 462 | 4.33 | 18 | -76 | -12 |
|  | Fusiform gyrus | 37 | — | 3.79 | 28 | -66 | -8 |
|  | Visual association area | 19 | 375 | 3.93 | 18 | -68 | 28 |
|  | Amygdala | — | 777 | 4.03 | -18 | 0 | -22 |
| AA homozygous BD patients < AA homozygous controls | Auditory association area | 22 | 363 | 4.47 | -50 | -14 | -4 |
|  | Primary auditory cortex | 41 | — | 4.46 | -48 | -18 | 10 |
| G allele carrier BD patients > G allele carrier controls | Anterior cingulate cortex | 32 | 155 | 4.68 | 6 | 44 | 6 |
|  | Insula | 13 | 158 | 3.95 | 42 | -8 | 6 |
|  | Visual association area | 18 | 403 | 4.31 | 16 | -90 | 22 |
|  | Primary visual cortex | 17 | — | 3.88 | 26 | -68 | 12 |
|  | Visual association area | 19 | 640 | 4.17 | -34 | -78 | -6 |
| G allele carrier BD patients *vs*. AA homozygous BD patients | NA | NA | NS | NS | NA | NA | NA |

Peak coordinates refer to the Montreal Neurological Institute space. Significance was thresholded at the uncorrected voxel level *p*=0.001, followed by the FWE-corrected cluster level *p*=0.05. BA, Brodmann area; BD, bipolar disorder; NA, not available; NS, not significant.**Supplementary Table S5.**

Peak MNI coordinates of the regions exhibiting significant resting-state functional connectivity with the left and right dorsal striatum with between-rs2228485 genotype and between-group differences

|  |  |  |  |  |  | Peak coordinate | | |
| --- | --- | --- | --- | --- | --- | --- | --- | --- |
| Lateral | Contrast | Region | BA | Cluster | *Z* score | *x* | *y* | *z* |
| **Left** | AA homozygous controls > G allele carrier controls | Primary auditory cortex | 41 | 258 | 3.80 | 62 | -10 | 0 |
|  |  | Auditory association area | 22 | — | 3.57 | 64 | 2 | -8 |
|  | G allele carrier BD patients *vs*. AA homozygous BD patients | NA | NA | NS | NS | NA | NA | NA |
| **Right** | AA homozygous controls > G allele carrier controls | Auditory association area | 22 | 198 | 3.99 | 64 | 0 | -10 |
|  |  | Primary auditory cortex | 41 | — | 3.85 | 64 | -20 | 6 |
|  |  | Visual association area | 19 | 372 | 4.12 | -44 | -78 | -12 |
|  |  | Visual association area | 19 | 469 | 3.88 | 42 | -82 | -18 |
|  | G allele carrier BD patients *vs*. AA homozygous BD patients | NA | NA | NS | NS | NA | NA | NA |
|  | (AA homozygotes > G allele carriers) (controls > BD patients) | Visual association area | 19 | 321 | 4.68 | -44 | -78 | -12 |
|  |  | Visual association area | 18 | 286 | 4.15 | 34 | -94 | -2 |

Peak coordinates refer to the Montreal Neurological Institute space. Significance was thresholded at the uncorrected voxel level *p*=0.001, followed by the FWE-corrected cluster level *p*=0.05. BA, Brodmann area; BD, bipolar disorder; NA, not available; NS, not significant.

**Supplementary Table S6.**

Peak MNI coordinates of the regions exhibiting significant resting-state functional connectivity between-rs2228485 genotype and between-group differences in a euthymic-only sample

|  |  |  |  |  |  |  | Peak coordinate | | |
| --- | --- | --- | --- | --- | --- | --- | --- | --- | --- |
| Contrast | Seed | Lateral | Region | BA | Cluster | *Z* score | *x* | *y* | *z* |
| (AA homozygotes > G allele carriers) (controls > BD patients) | Dorsal striatum | Right | Visual association area | 19 | 197 | 4.60 | -46 | -76 | -12 |
|  | Ventral striatum | Right | Primary auditory cortex | 41 | 206 | 4.95 | -46 | -14 | 8 |
|  |  |  | Auditory association area | 22 | — | 4.04 | -56 | -22 | 0 |
|  |  |  | Primary somatosensory cortex | 1 | 226 | 4.34 | 48 | -10 | 10 |
|  |  |  | Insula | 13 | — | 4.33 | 34 | -18 | 10 |
|  |  |  | Primary auditory cortex | 41 | — | 3.12 | 44 | -28 | 12 |
| AA homozygous controls > G allele carrier controls | Ventral striatum | Left | Auditory association area | 22 | 151 | 3.99 | 50 | -38 | 10 |
|  |  |  | Primary auditory cortex | 41 | 277 | 3.96 | -44 | -10 | 10 |
|  |  |  | Secondary somatosensory cortex | 40 | — | 3.81 | -56 | -24 | 10 |
|  |  |  | Visual association area | 19 | 479 | 4.23 | 24 | -80 | -10 |
|  |  |  | Fusiform gyrus | 37 | — | 3.79 | 28 | -66 | -12 |
|  |  |  | Visual association area | 19 | 245 | 3.82 | -52 | -74 | -6 |
|  |  |  | Hippocampus | — | 142 | 4.67 | 28 | -12 | -16 |
|  |  |  | Amygdala | — | — | 4.04 | 26 | -4 | -18 |
|  |  |  | Amygdala | — | 155 | 3.75 | -24 | -4 | -20 |
|  |  |  | Primary somatosensory cortex | 1 | 332 | 3.99 | 52 | -20 | 44 |
|  |  |  | Insula | 13 | 228 | 3.87 | 42 | -6 | 2 |
|  |  |  | Primary auditory cortex | 41 | — | 3.48 | 40 | -22 | 14 |
|  |  |  | Supramarginal gyrus | 40 | 130 | 3.29 | 62 | -18 | 18 |
|  | Ventral striatum | Right | Primary auditory cortex | 41 | 851 | 4.89 | 68 | -8 | 0 |
|  |  |  | Insula | 13 | — | 4.53 | 42 | -8 | 8 |
|  |  |  | Primary auditory cortex | 41 | — | 4.32 | 40 | -20 | 12 |
|  |  |  | Hippocampus | — | 145 | 4.93 | 28 | -12 | -16 |
|  |  |  | Primary auditory cortex | 41 | 508 | 4.95 | -46 | -12 | 8 |
|  |  |  | Auditory association area | 22 | — | 4.39 | -62 | -30 | 16 |
|  |  |  | Visual association area | 19 | 179 | 4.15 | -32 | -74 | -16 |
|  |  |  | Visual association area | 18 | 322 | 3.97 | 18 | -76 | -12 |
|  |  |  | Fusiform gyrus | 37 | — | 3.70 | 28 | -66 | -14 |
|  |  |  | Visual association area | 19 | 263 | 3.65 | 18 | -68 | 28 |
|  |  |  | Orbitofrontal cortex | 11 | 321 | 3.39 | -14 | 30 | -20 |
|  | Dorsal striatum | Left | Primary somatosensory cortex | 1 | 348 | 3.90 | 58 | -24 | 50 |
|  | Dorsal striatum | Right | Visual association area | 19 | 371 | 4.02 | -44 | -78 | -12 |
|  |  |  | Primary auditory cortex | 41 | 260 | 3.98 | 64 | -4 | 0 |
|  |  |  | Visual association area | 19 | 291 | 3.73 | 24 | -76 | -8 |
|  |  |  | Fusiform gyrus | 37 | — | 3.15 | 28 | -70 | -12 |
| AA homozygous BD patients < AA homozygous controls | Ventral striatum | Left | Primary somatosensory cortex | 1 | 171 | 4.37 | -54 | -26 | 56 |
|  |  |  | Corpus callosum | — | 132 | 4.10 | 12 | 12 | 20 |
|  | Ventral striatum | Right | Primary auditory cortex | 41 | 376 | 4.43 | -48 | -14 | 8 |
|  |  |  | Hippocampus | — | 128 | 4.00 | 28 | -14 | -16 |
|  |  |  | Amygdala | — | — | 3.80 | 26 | -6 | -16 |
|  |  |  | Hippocampus | — | 134 | 3.64 | -24 | -16 | -14 |
| G allele carrier BD patients > G allele carrier controls | Ventral striatum | Left | Hippocampus | — | 133 | 4.18 | -24 | -40 | 2 |
|  | Ventral striatum | Right | Visual association area | 18 | 695 | 4.75 | 16 | -88 | 22 |
|  |  |  | Visual association area | 18 | 171 | 4.25 | -24 | -76 | -4 |
|  | Dorsal striatum | Left | Visual association area | 18 | 218 | 4.57 | 10 | -88 | 26 |
|  | Dorsal striatum | Right | Visual association area | 19 | 168 | 4.00 | -38 | -78 | -16 |
| G allele carrier BD patients *vs*. AA homozygous BD patients |  |  | NA | NA | NS | NS | NA | NA | NA |

Peak coordinates refer to the Montreal Neurological Institute space. Significance was thresholded at the uncorrected voxel level *p*=0.001, followed by the FWE-corrected cluster level *p*=0.05. BA, Brodmann area; BD, bipolar disorder; NA, not available; NS, not significant.

**Supplementary Table S7.**

Functional connectivity of ventral and dorsal striatum co-varying with depression in bipolar patients.

|  |  |  |  |  |  |  |  |  | Peak coordinate | | |
| --- | --- | --- | --- | --- | --- | --- | --- | --- | --- | --- | --- |
| Seed | Lateral | Group | Genotype | Direction | Region | BA | Cluster | *Z* score | *x* | *y* | *z* |
| Ventral striatum | Right | BD patients | AA homozygotes | Positive | Primary auditory cortex | 41 | 158 | 3.93 | 62 | -6 | 0 |
|  |  |  |  |  | Auditory association area | 22 | — | 3.55 | 60 | -4 | -10 |
| Dorsal striatum | Left | BD patients | G allele carriers | Negative | Visual association area | 18 | 464 | 4.15 | -4 | -88 | -14 |
|  |  |  |  |  | Fusiform gyrus | 37 | 121 | 4.73 | 40 | -38 | -18 |
|  |  |  |  |  | Primary somatosensory cortex | 1 | 144 | 3.69 | -10 | -34 | 74 |
|  |  |  |  |  | Primary somatosensory cortex | 1 | 122 | 3.37 | 48 | -8 | 12 |
|  |  |  |  |  | Primary motor cortex | 4 | 124 | 5.37 | -54 | -6 | 14 |
|  |  |  |  |  | Primary motor cortex | 4 | 2774 | 4.85 | 6 | -28 | 66 |
|  | Right | BD patients | G allele carriers | Negative | Primary somatosensory cortex | 1 | 747 | 4.08 | 20 | -34 | 74 |
|  |  |  |  |  | Sensory association area | 5 | 134 | 3.63 | 6 | -36 | 56 |
|  |  |  |  |  | Cerebellum | — | 375 | 4.77 | -32 | -84 | -22 |

One bipolar patient with G allele and 1 control with G allele did not complete the 17-item Hamilton Depression Rating Scale (HDRS) and were excluded from this calculation. Peak coordinates refer to the Montreal Neurological Institute (MNI) space. Significance was thresholded at the uncorrected voxel level *p*=0.001, followed by the FWE-corrected cluster level *p*=0.05. No correlation was found in AA homozygous controls. BA, Brodmann area; BD, bipolar disorder.

**Supplementary Table S8.**

Functional connectivity of ventral and dorsal striatum co-varying with loneliness.

|  |  |  |  |  |  |  |  |  | Peak coordinate | | |
| --- | --- | --- | --- | --- | --- | --- | --- | --- | --- | --- | --- |
| Seed | Lateral | Group | Genotype | Direction | Region | BA | Cluster | *Z* score | *x* | *y* | *z* |
| Ventral striatum | Left | Controls | AA homozygotes | Negative | Posterior parietal lobe | 7 | 186 | 4.44 | -34 | -58 | 56 |
|  |  | BD patients | AA homozygotes | Negative | Anterior cingulate cortex | 24 | 159 | 3.56 | 4 | 34 | 12 |
|  |  |  |  |  | Cerebellum | — | 151 | 4.34 | -20 | -82 | -30 |
|  |  | BD patients | G allele carriers | Negative | Posterior parietal lobe | 39 | 206 | 4.25 | -38 | -52 | 34 |
|  |  |  |  |  | Posterior parietal lobe | 39 | 213 | 3.96 | 30 | -70 | 44 |
|  |  |  |  |  | Posterior parietal lobe | 7 | 176 | 3.88 | 0 | -76 | 46 |
|  |  |  |  |  | Premotor cortex | 6 | 167 | 4.21 | -22 | 10 | 50 |
|  | Right | BD patients | G allele carriers | Negative | Posterior parietal lobe | 39 | 247 | 4.54 | -32 | -54 | 40 |
|  |  |  |  |  | Posterior parietal lobe | 39 | 230 | 4.24 | 36 | -68 | 30 |
|  |  |  |  |  | Ventrolateral prefrontal cortex | 44 | 850 | 4.37 | -40 | 22 | 18 |
|  |  |  |  |  | Ventrolateral prefrontal cortex | 44 | 209 | 3.74 | 58 | 18 | 24 |
|  |  |  |  |  | Premotor cortex | 6 | 227 | 3.88 | -38 | 8 | 56 |
| Dorsal striatum | Left | BD patients | AA homozygotes | Negative | Cerebellum | — | 119 | 3.91 | -10 | -56 | -44 |
|  |  | BD patients | G allele carriers | Negative | Frontal eye fields | 8 | 183 | 3.73 | -4 | 20 | 52 |
|  | Right | BD patients | AA homozygotes | Negative | Visual association area | 19 | 130 | 4.54 | -44 | -82 | 2 |
|  |  | BD patients | G allele carriers | Negative | Ventrolateral prefrontal cortex | 10 | 169 | 3.88 | 20 | 62 | 14 |

Two bipolar patients and 1 control did not complete the loneliness and were excluded from this calculation. Peak coordinates refer to the Montreal Neurological Institute (MNI) space. Significance was thresholded at the uncorrected voxel level p=0.001, followed by the FWE-corrected cluster level p=0.05. No correlation was found in G allele carrier controls. BA, Brodmann area.
